# Evaluating effects of selected water conservation techniques and manure on sorghum yields and rainwater use efficiency in dry region of Zimbabwe

**DOI:** 10.1016/j.heliyon.2024.e33032

**Published:** 2024-06-14

**Authors:** Andrew Tapiwa Kugedera, Letticia Kudzai Kokerai, George Nyamadzawo, Ronald Mandumbu

**Affiliations:** a Department of Environmental Science, Bindura University of Science Education, P. Bag 1020, Bindura, Zimbabwe; b Department of Crop and Livestock, Ministry of Lands, Agriculture, Fisheries, Water and Rural Development, Masvingo, Zimbabwe; c Department of Crop Science, Bindura University of Science Education, P. Bag 1020, Bindura, Zimbabwe

**Keywords:** Rainwater use efficiency, Tied contour, Semi-arid areas, Sorghum productivity

## Abstract

Sorghum production in semi-arid areas of Zimbabwe is constrained by low and erratic rainfall, low fertility and soil moisture stress. Sorghum grain yields ranges from 0.2 to 0.4 t ha^−1^ in sandy-to-sandy loam soils respectively. The objective of the study was to assess cattle manure and rainwater harvesting techniques in improving sorghum grain yield in a semi-arid region of Zimbabwe. The experiment used a randomised complete block design with rainwater harvesting technique as a main treatment factor at three levels. Sub-plot factor was cattle manure at five levels (0, 2.5, 5, 10 and 15 t ha^−1^) and two sorghum varieties (Macia and SV1) as sub-sub plot factor. Sorghum grain yields were improved significantly (p < 0.05) for both varieties using tied contours. Increasing application rates of cattle manure, showed significant increase (p < 0.05) in sorghum grain yield over the control (0 t ha^−1^). Tied contour had higher grain yield (1.15 t ha^−1^) with the use of Macia variety. Stover yield was highly influenced by rainwater harvesting method of tied contour (p < 0.05) compared with infiltration pit and standard contour. Increase in application levels of cattle manure show significant (p < 0.05) increase in stover yields. Tied contour had the highest (3.11 kg ha^−1^ mm^−1^) rainwater use efficiency which show significant differences (p < 0.05) from infiltration pits and standard contour. Interaction of tied contour and different rates of cattle manure showed significant increments in rainwater use efficiency with increases in manure application rates. Tied contours, 15 t ha^−1^ cattle manure and Macia variety are potential strategy to achieve climate smart agriculture and improve food security in semi-arid areas. Sorghum production in marginalised areas can be productive with adoption of tested techniques.

## Highlights

• Sorghum production is constrained by low rainfall and soil infertility in semi-arid areas.
• Tied contour and infiltration pits have potential to improve soil moisture and sorghum yield.
• Interaction of cattle manure with tied contour increase rainwater use efficiency and sorghum yield.

## 1 Introduction

Land degradation and unreliable rainfall in semi-arid areas influences crop production under rainfed agriculture [1,2]. Land degradation is caused by high rainfall intensity and lack of soil cover especially in semi-arid areas dominated by sandy-to-sandy loam soils [[3], [4], [5]]. Smallholder farmers in semi-arid areas have limited knowledge on proper water and soil fertility management [2,6,7]. Sorghum grain yield under rainfed agriculture in smallholder farming environments has been low (<0.3 t ha^−1^) [8], increasing risk of food insecurity and poverty [[9], [10], [11]]. Unreliable and erratic rainfall cause frequent droughts and mid-season dry spells which affect important plant physiological stages such as grain filling and kernel development leading to low yield [1,4,12,13]. Adoption of sorghum, field edge rainwater harvesting such as infiltration pits (IP) and tied contours (TC) improve soil water content and reduce food insecurity in smallholder farming environments. Most farmers have been previously using standard contours which dispose-off rainwater and reduce sorghum yield. There is need for farmers to use permanent rainwater harvesting methods like IP and TC which capture rainwater, reduce flooding in fields, soil erosion and increase soil moisture content during cropping season [2,10,14].

Sorghum (*Sorghum bicolor* (L. Moench)) is among important cereal crops globally [15] and is ranked second best to maize in sub-Saharan African (SSA) countries such as Kenya [9], Tanzania [16] and Zimbabwe [5]. Sorghum performs better than maize under harsh environmental conditions and can be a good option to increase food security in semi-arid areas [7,17]. Sorghum performs better in marginalised areas associated with low rainfall and high temperatures [[17], [18], [19]]. Sorghum productivity can be improved through soil and moisture management options [10,11,20,21] and become one of the best staple foods in Africa [16,22]. In Zimbabwe sorghum grain yield averages at 0.25–0.3 t ha^−1^ [8,23,24] and increased to 0.8 t ha^−1^ with the use of water and nutrient management in sandy loam soils under smallholder farming systems [11].

Tied contour is a variation of standard contour, which has been modified to capture rainwater, reduce surface runoff and recharge soil moisture content [11]. Tied contours were mainly evaluated in Zimbabwe and show positive effects on soil moisture content for example in Shurugwi [25] and Chivi [5,11], improve crop yields in Mutare [2,10,14], Shurugwi [25] and Chivi [11,26].

Infiltration pits were developed in Zvishavane, Zimbabwe to capture rainwater and increase soil moisture content in sandy loam soils which have low water retention capacity [5,27]. Adoption of infiltration pits in low rainfall areas of Africa was reported to improve crop yields in countries such as Burkina Faso [28], Tanzania [16,29] and Zimbabwe [2,6,11,14,19,25]. Use of infiltration pits has been reported to collect more water which can be used later by crops [14,25,29,30], mitigate total crop failure caused by long mid-season droughts especially in the dry regions of SSA [1,16,31] and increase soil water content [6,7,13,19,25,30,32,33] especially in soils with low water retention capacity. The use of rainwater harvesting alone address soil moisture stress leaving issues of soil fertility hanging, hence there is need to adopt the use of organic manure readily available in smallholder farming environments such as cattle manure. This will create a positive synergistic association by making soil water and nutrients readily available to crops hence increase sorghum grain yield [9].

Cattle manure improve soil structure, water retention capacity, regulate soil pH and increase soil nutrient content in plant rooting zone [13,19]. Application of cattle manure was reported to increase sorghum grain yield from 0.3 t ha^−1^ to around 1 t ha^−1^ in areas where soil have been heavily depleted [5,34,35]. According to Ref. [36] in Kenya and [37] in Zimbabwe, cattle manure is a cheap source for poor resource farmers in SSA countries and can be used in large quantities compared to inorganic fertilisers [13,19,35]. In Zimbabwe [37] used large quantities of 12.5 and 37.5 t ha^−1^ in maize [38], used 15 and 30 Mg in tomato [39], used 5 to 25 t ha^−1^ for maize production in Murehwa and [40] used 5 to 15 t ha^−1^ in smallholder farming areas of Domboshava to improve rape production. Recommended quantities of cattle manure are 20–40 t ha^−1^ which translate to 200–440 kg N ha^−1^ and this becomes unrealistic to farmers due to several factors. Lowering quantities of cattle manure to 2.5–15 t ha^−1^ makes it easy for its utilisation by smallholder farmers. Previous studies by Ref. [23] show that the use of cattle manure and ridges in sorghum production can improve sorghum grain yield to 4 t ha^−1^. This yield has been too low and can be increased with the use of improved field edge rainwater harvesting techniques such as tied contours which has the capacity to harvest and store water which plants can use during dry spell. The objective of the study was to evaluate the effects of tied contour, infiltration pits and different rates of cattle manure on sorghum yields and rainwater use efficiency of two sorghum varieties in dry region of Zimbabwe. The specific hypothesis being tested was that the tied contour and manure can significantly increase sorghum productivity.

## 2 Materials and methods

### 2.1 Experimental site

Field experiment was carried out from 2017/18 to 2019/2020 growing seasons in a dry area in Chivi district (20°13.24.8′S and 30°28.33.2′E, 924 masl), 78 km from Masvingo town in the south-western part of Zimbabwe. The site is in natural farming region IV which receive rainfall ranging from 300 to 450 mm (averages at 335 mm) [26]. Average annual temperature of the area is 25 °C and associated with cold temperatures in the month of June and July. Soils in the experimental field were sandy loam with low nitrogen content and moderately fertile [11]. Rainfall was poorly distributed and associated with mid-season dry spell. The rainfall commences in mid-November and ends in March. Rainfall distribution was uneven and associated long mid-season dry spells.

### 2.2 Soil characteristics

Soil sampling and testing was done every cropping season before land preparation. Fifteen samples of soil were collected randomly and thoroughly mixed to form a composite sample (1 kg) for analysis. A 2 mm sieve was used to remove stones and other unwanted materials and analysed for pH by Calcium chloride (CaCl_2_) method [26,41], soil texture was determined using Bouyoucos hydrometer method [26,42], total nitrogen by Kjeldahl method [7,43], soil organic carbon by wet digestion method and available phosphorous by Olsen method [7,44]. Ammonium acetate methods at pH of 7 were used to determine exchangeable potassium, calcium and magnesium [7,41].

## 3 Experimental materials

The variety of sorghum used for this study was the Macia and SV1 which were obtained from Grain Marketing Board (GMB). These are open pollinated varieties with an average of 115 days to maturity [26]. SV1 is a semi-short seasoned variety with mean height of 125–180 cm depending on ecological region. Macia is a short-seasoned variety with an average plant height of 120–150 cm and gives a yield ranging from 0.2 to 0.4 t ha^−1^ in smallholder farming environments. SV1 yield ranges between 3 and 6 t ha^−1^ under commercial farming and averages at 0.25 t ha^−1^ under smallholder farming systems. Cattle manure was obtained from local farmers.

### 3.1 Experimental design and treatments

**Fig. 1:**
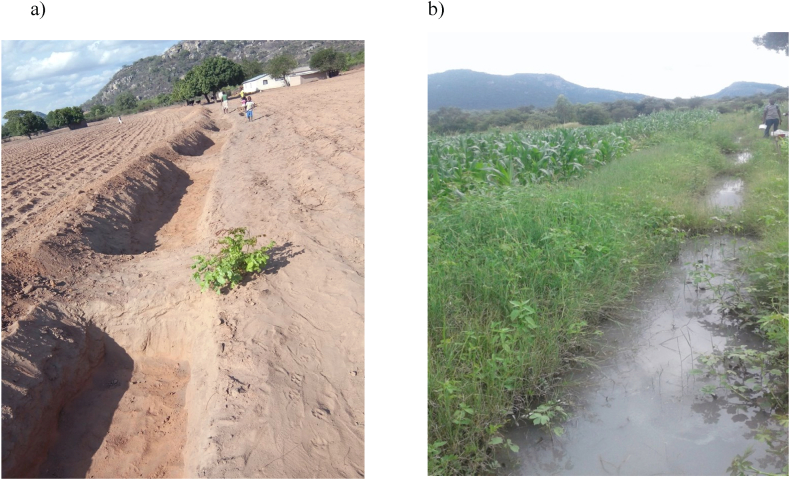
Tied contour a) before rainwater collection and b) after rainwater collection.

**Fig. 2:**
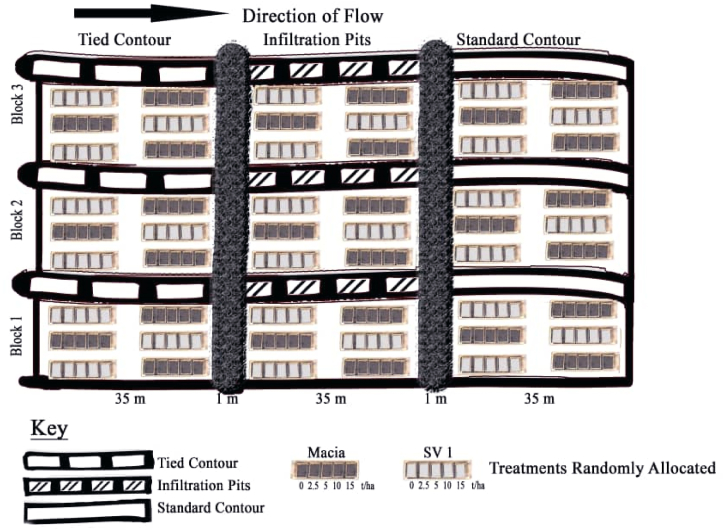
Experimental field layout.

Experimental treatments were arranged in a randomised complete block design with treatment factors replicated three times. Rainwater harvesting method was the main treatment factor with three levels: infiltration pits (0.5 m wide x 0.5 m deep x 3 m long) along a 36 m long contour, tied contour (0.5 m × 0.5 m x 3 m long) for 36 m long (Fig. 1a and b) and standard contour of 0.5 m wide x 0.5 m deep x 36 m long. Sub plot factor was cattle manure with five levels (0, 2.5, 5, 10 and 15 t ha^−1^) and randomly allocated. Test crop (sorghum) was used as fixed sub-sub plot factor with two levels (Macia and SV1 varieties) as indicated on Fig. 2. Treatments were randomly allocated to treatment plots measuring 3 m × 4.5 m. Exact cattle manure applied per plot (13.5 m^2^) for 0, 2.5, 5, 10 and 15 t ha^−1^ were 0, 33.75, 67.5, 135 and 202.5 kg respectively. Plant spacing between rows of 0.8 m and 0.15 m in row to achieve plant population of 83333 plants ha^−1^ with one plant per stand. Animal drawn mouldboard plough was used to achieve a depth of 20 cm on average. Furrows for planting were opened with hand hoe after marking sizes of all experimental plots. Cattle manure was applied three months before planting to allow for decomposition and release of nutrients. Cattle manure was applied when soil moisture content was at an average of 1.5 % before planting (on July 18, 2017, July 10, 2018 and August 10, 2019) and no rainfall was received during this period. Chemical composition of cattle manure used during the experiment is shown in Table 1. Sorghum seeds were sown on November 18, 2017, November 10, 2018 and December 10, 2019 for 2017/18, 2018/19 2019/20 cropping seasons respectively. Thinning was done four weeks after germination to leave one plant per stand. Weeding was done same time as thinning using hand hoe. Ammonium nitrate (34.5 % N) was applied three weeks after planting at a rate of 100 kg ha^−1^.

**Table 1:** Chemical composition of cattle manure used during the experimental period cattle manure.

Component	2017/18	2018/19	2019/20
Nitrogen (g kg^−1^)	11	13	10.8
Total Carbon (g kg^−1^)	112	112	112
Available P (g kg^−1^)	2	3.4	2.6
Exchangeable Mg (g kg^−1^)	4	3.8	4.2
Exchangeable Ca (g kg^−1^)	9	9.1	8.9
Available K (g kg^−1^)	18	17.6	16
Moisture content (%)	17	15	19

### 3.2 Rainfall

**Fig. 3:**
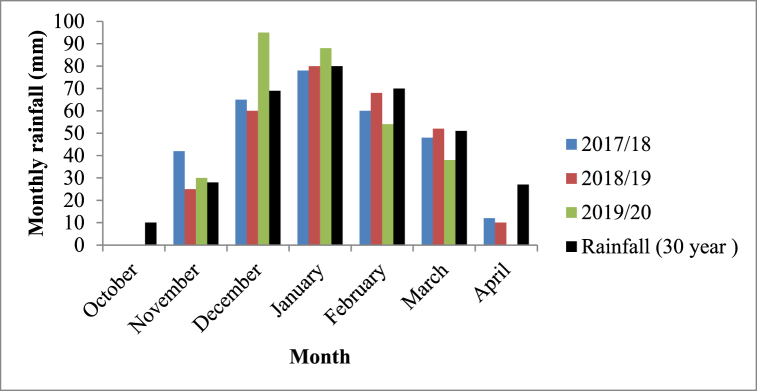
Monthly totals of rainfall received at the study during 2017/18 to 2019/20 season and 30-year monthly average (in black colour). (For interpretation of the references to colour in this figure legend, the reader is referred to the Web version of this article.)

Rainfall was measured using a standard rain gauge installed in the experimental site. Total rainfall received from 2017/18 to 2019/20 was lower than 30-year seasonal mean of 335 mm (Fig. 3). Highest rainfall was received in December during 2019/20 cropping season. Rainfall received during the experimental period was associated with frequent seasonal dry spells. Rainfall received was low in November and insignificant amounts received in April (Fig. 3).

### 3.3 Data collection

Data collected was based on rainfall, rainwater use efficiency, grain and stover yields. A standard rain gauge was used to collect daily rainfall. Sorghum yields (grain and stover) were harvested from a net plot measuring 3 m × 3 m. Panicles in the net plot were cut and sun dried to achieve grain moisture content 12 %. After threshing, weight was recorded for each treatment and its replicates for both varieties. Stover was cut closer to ground and chopped into smaller pieces to allow easy weighing. Cutting and threshing was done manually. Sorghum yields were converted to t ha^−1^ using formula below:

$$\text{Yield}\;\left(t\;{\text{ha}}^{‐1}\right)=\frac{Yield\;in\;the\;net\;plot\times 10000m^{2}}{net\;plot\;area}$$

where: 10000 m^2^ equals 1 ha and net plot = 9 m^2^.

### 3.4 Rain water use efficiency (RWUE)

This is the efficiency in which rainfall is converted to grain. This was calculated after harvesting of grain using the total grain yield per treatment and total rainfall received per season [7,26].

$$\text{RWUE}\;\left(\text{kg}\;{\text{ha}}^{‐1}\;{\text{mm}}^{‐1}\right)=\frac{\text{Total}\;\text{grain}\;\text{yield}\;\left(\;\text{kg}\;{\text{ha}}^{‐1}\right)\;}{\text{Total}\;\text{rainfall}\;\left(\text{mm}\right)}$$

### 3.5 Statistical data analysis

Collected data was entered into Microsoft excel for easy transformation and analysis of variance (ANOVA) was performed using GenStat 14th edition. Fisher's least significance difference (LSD_0.05_) was used to separate significant means (p ≤ 0.05).

## 4 Results

### 4.1 Soil characteristics

Table 2 summarises the soil physiochemical properties at study area. Results from initial analysis show that the soil had 85 % sand, 9 % silt and 6 % clay. Average soil pH was 5.2 with 1.1 g kg^−1^ total nitrogen, 10.7 g kg^−1^ soil organic carbon, 3.46 mg kg^−1^ phosphorous and 0.31 cmol_(+)_kg^−1^ potassium. Exchangeable calcium and magnesium were 0.96 cmol_(+)_kg^−1^ and 0.42 cmol_(+)_kg^−1^ respectively. Soil chemical parameters were increased by application of cattle manure (Table 2).

**Table 2:** Physiochemical properties of soil at study area.

Parameter	Composition	
	2017/18 (initial)	2019/20 (Final)
pH	5.2	5.3
SOC g kg^−1^	10.7	12.2
Total Nitrogen g kg^−1^	1.1	1.1
P_2_O_5_ mg kg^−1^	3.46	4.89
K_2_O cmol (+)kg^−1^	0.31	0.42
Calcium cmol (+)kg^−1^	0.96	1.1
Magnesium cmol (+)kg^−1^	0.42	0.512

#### 4.1.1 Effects of variety, RWH techniques, cattle manure and season on sorghum yields and rainwater use efficiency

Sorghum grain yield was significantly (p < 0.05) affected by sorghum variety, RWH techniques, application rate of cattle manure and season (Table 3). Sorghum grain yields were better from Macia variety, tied contour (TC), 15 t ha^−1^ of cattle manure and 2018/19 cropping season. Increasing application rate of cattle manure by 2.5 t ha^−1^ increased sorghum grain yields by 13.3 % and this was the highest increment across all application rates (Table 3).

**Table 3:** Effects of main treatment factors on sorghum yields and RWUE.

Treatments	Grain yields (t ha^−1^)	Stover yield (t ha^−1^)	RWUE (kg ha^−1^mm^−1^)
**Variety**			
Macia	0.823^a^	2.18^a^	2.95
SV1	0.776^b^	2.04^b^	2.72
**P value**	**<0.001**	**<0.001**	**<0.001**
LSD (0.05)	0.022	0.013	0.057
**Cattle manure (t ha**^**−**^**^1^)**			
0	0.725^e^	2.4^e^	2.49^e^
2.5	0.822^d^	2.45^d^	2.7^d^
5	0.888^c^	2.53^c^	2.94^c^
10	0.967^b^	2.55^b^	3.17^b^
15	1.09^a^	2.57^a^	3.41^a^
**P value**	**<0.001**	**<0.001**	**<0.001**
LSD (0.05)	0.043	0.009	0.073
**RWH**			
Infiltration pit (IP)	0.737^b^	2.31^b^	2.95^b^
Tied contour (TC)	0.788^a^	2.39^a^	3.11^a^
Standard contour (SC)	0.682^c^	2.22^c^	2.76^c^
**P value**	**<0.001**	**<0.001**	**<0.001**
LSD (0.05)	0.028	0.013	0.057
**Season**			
2018	0.885^b^	2.21	2.98
2019	0.979^a^	2.52	3.16
2020	0.835^c^	2.14	2.72
**P value**	**<0.001**	**<0.001**	**<0.001**
LSD (0.05)	0.043	0.009	0.073
CV (%)	3.2	2.1	2.8

Same superscripts in same column denotes no significant different between treatments at p ≤ 0.05 using Fisher's Least significant difference.

Sorghum stover yield was affected in the same manner as grain yields with better yields obtained from Macia variety, TC, 15 t ha^−1^ of cattle manure and 2018/19 cropping season. Sorghum stover yields increased significantly (p < 0.05) with application rates of cattle manure. Rainwater use efficiency (RWUE) followed a trend Macia > SV1; IP<TC>SC; 0 < 2.5<5 < 10<15 t ha^−1^ cattle manure, and 2018<2019>2020. Standard contour (SC) had the lowest RWUE which was significantly (p < 0.05) different from IP and TC treatment (Table 3). Sorghum yields and RWUE varied significantly with season, and were lowest in the 2019/20 season.

#### 4.1.2 Effects of RWH methods and season on grain yields

**Fig. 4:**
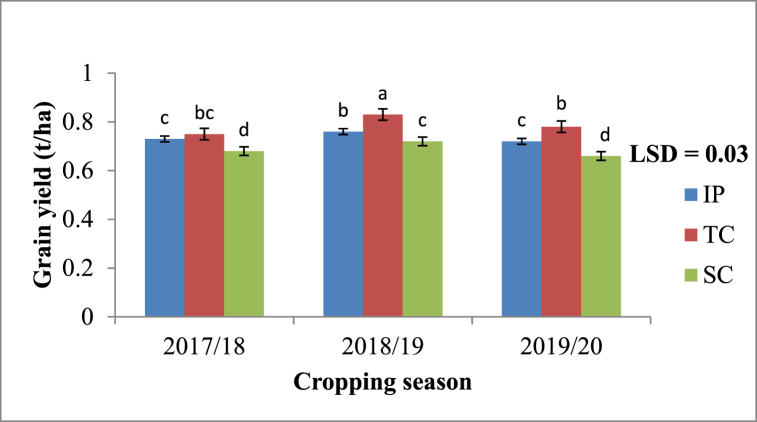
Effects of tied contour (TC), infiltration pit (IP) and standard contour (SC) on grain yield during 2017/18–2019/20. Bars with same superscript letter means no significant different at p ≤ 0.05 using Fisher's Least significant difference.

Grain yields were influenced (p < 0.05) by RWH techniques and season. Treatments with TC had the highest grain yields throughout the three cropping seasons (Fig. 4). Sorghum grain yields from TC were significantly higher (p < 0.05) compared to IP and SC over three seasons (Fig. 4). Standard contour had the lowest sorghum grain yields over three experimental seasons as compared to TC and IP.

#### 4.1.3 Effects of RWH techniques, season and variety on grain yields

**Fig. 5:**
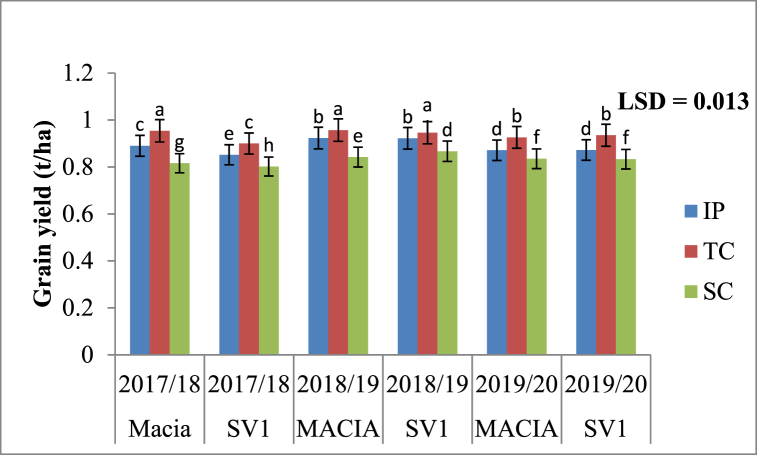
Effects of rainwater harvesting (RWH), season and sorghum variety grain yield during 2017/18 to 2019/20. IP = Infiltration pit; TC = Tied contour and SC = Standard contour. Bars with same superscript letter means no significant different at p ≤ 0.05 using Fisher's Least significant difference.

Grain yields show a trend: TC > IP > SC for both Macia and SV1 varieties. The effects of RWH techniques show significant (p < 0.05) increase on grain yields with higher yields observed from TC over all cropping seasons (Fig. 5). Highest grain yield (0.957 t ha^−1^) was from treatments with TC + Macia in 2018/19 season. Sorghum variety SV1 performed poorly on all treatments under SC. SV1 variety had significantly (p < 0.05) low grain yields during 2017/18 season for all RWH techniques.

The application of cattle manure shows significant (p < 0.05) improvements on sorghum grain yield (Table 4). Grain yields of 0.673 t ha^−1^ was observed from control treatments under SV1 variety and a higher grain yield of 1.048 t ha^−1^ from SV1 at 15 t ha^−1^ of cattle manure in 2017/18. The highest yield from SV1 variety was not significantly different (p > 0.05) with 1.054 t ha^−1^ from treatments with 15 t ha^−1^ cattle manure + Macia variety (Table 4). Application of cattle manure show significant increase (p < 0.05) in grain yields in 2018/19 compared to 2017/18 but decreased in 2019/20 for both varieties showing a trend: 2018 < 2019 >2020.

**Table 4:** Effects of cattle manure, season and sorghum variety on grain yields of sorghum during 2017–2020.

Cattle manure (t ha^−1^)	Macia Grain yield (t ha^−1^)			SV1 Grain yield (t ha^−1^)		
	2017/18	2018/19	2019/20	2017/18	2018/19	2019/20
0	0.759^e^	0.775^e^	0.771^e^	0.673^e^	0.777^e^	0.747^e^
2.5	0.809^d^	0.847^d^	0.829^d^	0.737^d^	0.846^d^	0.805^d^
5	0.876^c^	0.899^c^	0.872^c^	0.86^c^	0.919^c^	0.883^c^
10	0.959^b^	0.962^b^	0.925^b^	0.973^b^	0.979^b^	0.96^b^
15	1.031^a^	1.054^a^	0.99^a^	1.048^a^	1.037^a^	1.007^a^
LSD	0.018	0.011	0.003	0.018	0.011	0.003
*P-value*	*<0.001*	*<0.001*	*<0.001*	*<0.001*	*<0.001*	*<0.001*

Means in the same column followed by the same superscript (a-e) are not significantly different at p ≤ 0.05 using Fisher's Least significant difference.

#### 4.1.4 Effects of cattle manure and RWH techniques on grain yields

Sorghum grain yield increased significantly (p < 0.05) with combined effects of cattle manure and RWH techniques for both varieties. Sorghum grain yield show significant increments (p < 0.05) with increase in application rates of cattle manure irrespective of season, RWH and sorghum variety. Treatments with no cattle manure were insignificant (p > 0.05) on grain yield although TC had higher yields (Table 5). Highest grain yield (1.15 t ha^−1^) was from treatments with TC +15 t ha^−1^ cattle manure + Macia. Sorghum variety SV1 higher grain yields in 2018/19 which was significantly (p < 0.05) higher than that from Macia under same treatments except for treatments 0 and 15 t ha^−1^ cattle manure (Table 5). SV1 performed better than Macia in all three seasons under tied and standard contours combined with 5, 10 and 15 t ha^−1^ cattle manure (Table 5).

**Table 5:** Interactive effects of RWH and cattle manure on grain yields of sorghum during 2017–2020.

Treatment combinationsRWH techniques	Cattle manure (t ha^−1^)	Mean Macia grain yield (t ha^−1^)			Mean SV1 grain yield (t ha^−1^)		
		2017/18	2018/19	2019/20	2017/18	2018/19	2019/20
Infiltration Pit	0	0.782^d^	0.784^ab^	0.754^a^	0.670^a^	0.786^b^	0.74^b^
	2.5	0.8^d^	0.884^c^	0.806^b^	0.714^ab^	0.86^b^	0.776^b^
	5	0.908^b^	0.916^d^	0.876^c^	0.854^c^	0.938^ab^	0.87^cd^
	10	0.964^ab^	0.988^f^	0.926^d^	0.948^d^	0.988^a^	0.962^ef^
	15	0.998^ab^	1.044^g^	0.994^e^	1.074^f^	1.038^a^	1.014^g^
Tied Contours	0	0.798^d^	0.808^b^	0.882^c^	0.678^a^	0.804^b^	0.808^c^
	2.5	0.9^c^	0.884^c^	0.884^c^	0.790^bc^	0.874^b^	0.888^d^
	5	0.908^b^	0.942^e^	0.912^d^	0.952^d^	0.964^a^	0.94^ef^
	10	1.026^a^	1.004^fg^	0.968^de^	0.964^de^	1.006^a^	1.01^g^
	15	1.14^a^	1.146^g^	1.024^e^	1.116^f^	1.08^a^	1.028^g^
Standard Contour	0	0.698^f^	0.734^a^	0.718^a^	0.672^a^	0.74^b^	0.692^a^
	2.5	0.726^e^	0.774^a^	0.796^b^	0.706^ab^	0.804^b^	0.75^b^
	5	0.812^d^	0.838^bc^	0.828^bc^	0.774^abc^	0.856^b^	0.838^cd^
	10	0.888^c^	0.894^c^	0.882^c^	0.900^cd^	0.942^ab^	0.908^e^
	15	0.954^ab^	0.972^f^	0.95^de^	0.956^d^	0.994^a^	0.978^f^
LSD (5 %)		0.042	0.028	0.089	0.042	0.028	0.089
*P-value*		0.001	0.012	<0.001	0.001	0.012	<0.001
CV (%)		2.8	1.9	2.2	2.8	1.9	2.2

Means in the same column followed by the same superscript (a-g) are not significantly different at p ≤ 0.05 using Fisher's Least significant difference.

### 4.2 Stover yields

**Fig. 6:**
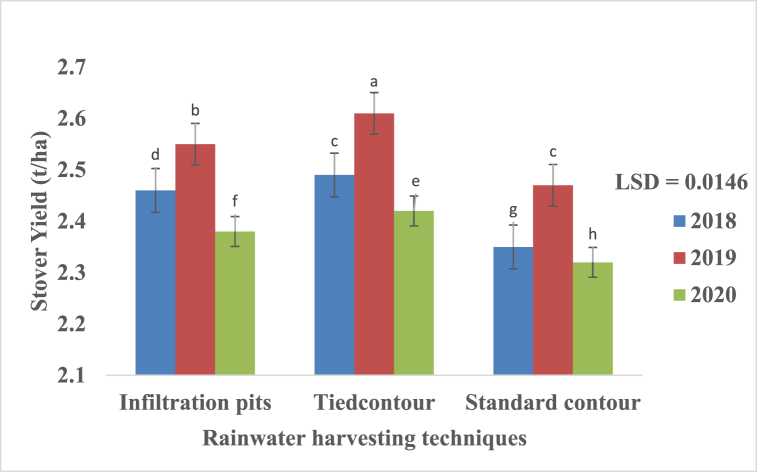
Effects of rainwater harvesting techniques on stover yield from 2017/18 to 2019/20 cropping season. Bars with same superscript letter means no significant different at p ≤ 0.05 using Fisher's Least significant difference.

Stover yield was significantly (p < 0.05) influenced by different RWH techniques with SC having the lowest yield compared to IP and TC (Fig. 6). Tied contour had the best stover yield throughout three cropping seasons and yields show a trend: SC < IP < TC and 2018<2019>2020.

#### 4.2.1 Interactive effects of RWH, variety and season on stover yields

**Fig. 7:**
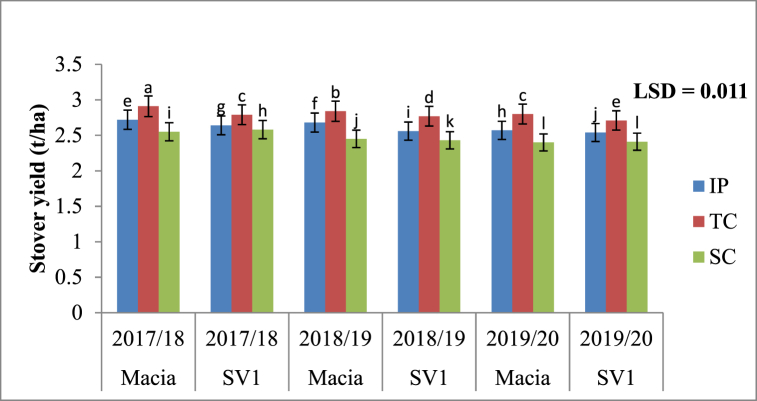
Interactive effects of rainwater harvesting (RWH), variety and season on stover yields of sorghum during 2017/18 to 2019/20. IP = Infiltration pit; TC = Tied contour and SC = Standard contour. Bars with same superscript letter means no significant different at p ≤ 0.05 using Fisher's Least significant difference.

Stover yield under three RWH techniques follow a trend: SC < IP < TC for two varieties Macia and SV1 across all three growing seasons. Tied contour had highest stover yield irrespective of season and variety which show significant (p < 0.05) differences when compared to IP and SC (Fig. 7). Stover yields were insignificant for all SC treatments across all seasons and varieties. The performance of IP on stover yield was also comparable throughout the three cropping seasons and two varieties (Fig. 7).

Stover yields increased significantly (p < 0.05) with increase in application rates of cattle manure and sorghum varieties in all three growing seasons. Macia performed at all different application rates of cattle manure in comparison to SV1 over three growing seasons (Table 6). The highest stover yield (2.564 t ha^−1^) was from treatments with Macia +15 t ha^−1^ cattle manure in 2017/18 and show significant (p < 0.05) differences from same treatments in 2018/19 and 2019/20. Macia performed significantly (p < 0.05) better than SV1 across all seasons and application rates of cattle manure.

**Table 6:** Effects of cattle manure and variety on sorghum stover yields of sorghum during 2017–2020.

Treatments	Mean Macia stover yield (t ha^−1^)			Mean	Mean SV1 stover yield (t ha^−1^)			Mean
Cattle manure (t ha^−1^)	2017/18	2018/19	2019/20		2017/18	2018/19	2019/20	
0	2.49^e^	2.47^c^	2.48^e^	2.48	2.428^e^	2.44^e^	2.394^e^	2.42
2.5	2.509^d^	2.48^c^	2.493^d^	2.49	2.442^d^	2.462^d^	2.421^d^	2.44
5	2.523^c^	2.5^b^	2.509^c^	2.51	2.471^c^	2.482^c^	2.451^c^	2.47
10	2.542^b^	2.52^a^	2.529^b^	2.53	2.5^b^	2.501^b^	2.489^b^	2.5
15	2.564^a^	2.528^a^	2.555^a^	2.55	2.518^a^	2.517^a^	2.515^a^	2.52
*P-value*	*<0.001*	*<0.001*	*<0.001*		*0.01*	*<0.001*	*<0.001*	
LSD (0.05)	0.011	0.009	*0.012*		0.011	0.009	*0.012*	

Means in the same column followed by the same superscript (a-e) are not significantly different at p ≤ 0.05 using Fisher's Least significant difference.

#### 4.2.2 Stover yields as influenced by RWH and cattle manure

Integrated effects RWH and cattle manure show significant (p < 0.05) increments in stover yields for two varieties (Macia and SV1) over three experimental seasons. Stover yields observed were better in 2017/18 from Macia over SV1 from all cattle manure application rates and RWH techniques. The best stover yield (2.93 t ha^−1^) was from treatments with TC + 15 t ha^−1^ + Macia in 2017/18 cropping season. Increasing application rates of cattle manure had significant (p < 0.05) effects on stover yields with higher increment observed when 5 t ha^−1^ was increased to 10 t ha^−1^ (Table 7). Stover yields were lowest from SC treatments from both varieties irrespective of season.

**Table 7:** Combined effects of RWH, cattle manure and variety on stover yields of sorghum during 2017–2020.

Treatments	Cattle manure (t ha^−1^)	Mean Macia stover yield (t ha^−1^)			Mean SV1 stover yield (t ha^−1^)		
RWH techniques		2017/18	2018/19	2019/20	2017/18	2018/19	2019/20
Infiltration Pit	0	2.305^b^	2.233^b^	2.21^a^	2.277^a^	2.227^a^	2.213^a^
	2.5	2.381^bc^	2.338^bc^	2.361^b^	2.357^b^	2.344^b^	2.300^b^
	5	2.465^c^	2.589^cd^	2.505^cd^	2.396^bc^	2.498^cd^	2.424^c^
	10	2.615^d^	2.666^d^	2.632^d^	2.517^c^	2.667^de^	2.546^d^
	15	2.844^e^	2.732^de^	2.728^de^	2.761^de^	2.736^e^	2.68^e^
Tied contour	0	2.329^b^	2.272^b^	2.3^b^	2.312^b^	2.279^b^	2.276^b^
	2.5	2.406^bc^	2.378^bc^	2.469^c^	2.358^b^	2.372^b^	2.446^c^
	5	2.590^cd^	2.653^d^	2.614^cd^	2.483^c^	2.617^d^	2.570^d^
	10	2.761^de^	2.75^de^	2.779^de^	2.601^cd^	2.727^e^	2.678^e^
	15	2.930^ef^	2.867^e^	2.885^e^	2.873^e^	2.834^f^	2.848^f^
Standard contour	0	2.212^a^	2.121^a^	2.15^a^	2.188^a^	2.143^a^	2.167^a^
	2.5	2.333^b^	2.198^a^	2.352^b^	2.315^b^	2.201^a^	2.208^a^
	5	2.429^c^	2.451^c^	2.460^c^	2.416^bc^	2.411^c^	2.335^b^
	10	2.550^cd^	2.57^cd^	2.576^cd^	2.539^cd^	2.509^cd^	2.475^c^
	15	2.731^de^	2.675^d^	2.674^d^	2.685^d^	2.639^d^	2.641^s^
LSD (5 %)		0.114	0.077	0.084	0.114	0.077	0.084
*P-value*		*<0.001*	*<0.001*	*<0.001*	*0.001*	*<0.001*	*<0.001*
CV (%)		*3.2*	*1.5*	*2.7*	*3.2*	*1.5*	*2.7*

Means in the same column followed by the same superscript (a-g) are not significantly different at p ≤ 0.05 using Fisher's Least significant difference.

### 4.3 Rainwater use efficiency (RWUE)

The use of RWH techniques had significant (p < 0.05) effects on RWUE over three experimental seasons. The results followed a trend TC > IP > SC with highest values obtained from tied contours throughout all three cropping seasons. Tied contour treatments had the highest (3.14 kg ha^−1^mm^−1^) three-year averages compared to infiltration pits and standard contour for both varieties (Table 8). Rainwater use efficiency increased by 10.3–14.5 % form Macia and by 8.3–10.9 % under SV1 varieties from standard contour to tied contour values respectively.

**Table 8:** Effects of RWH techniques on RWUE of sorghum during 2017–2020.

Treatments	Mean Macia RWUE (kg ha^−1^mm^−1^)			Mean	Mean SV1 RWUE (kg ha^−1^mm^−1^)			Mean
RWH	2017/18	2018/19	2019/20		2017/18	2018/19	2019/20	
Infiltration pit	2.92^b^	3.13^b^	2.86^b^	2.97	2.79^b^	3.13^b^	2.88^b^	2.93
Tied contour	3.13^a^	3.24^a^	3.04^a^	3.14	2.95^a^	3.21^a^	3.07^a^	3.07
Standard contour	2.67^c^	2.86^c^	2.74^c^	2.76	2.63^c^	2.94^c^	2.73^c^	2.77
*P-value*	*<0.001*	*<0.001*	*<0.001*		*<0.001*	*<0.001*	*<0.001*	
LSD (0.05)	0.095	0.06	0.015		0.095	0.06	0.015	

Means in the same column followed by the same superscript (a-c) are not significantly different at p ≤ 0.05 using Fisher's Least significant difference.

The use of cattle manure at different rates significantly (p < 0.05) influence RWUE (Table 9). Different application rates of manure applied significantly (p < 0.05) show effects on RWUE for both varieties and seasons. The magnitude of increase of RWUE over the control ranged from 6.1 to 26.4 % throughout the three seasons and two varieties. Effects of cattle manure on RWUE show a trend: 15 > 10>5 > 2.5>0 t ha^−1^ where a significant effect was noticed. Furthermore, three-year average RWUE for 0–15 t ha^−1^ cattle manure levels ranged from 2.55 to 3.4 kg ha^−1^mm^−1^ for Macia and 2.43–3.42 kg ha^−1^mm^−1^ for SV1 variety. However, RWUE increased with increase in cattle manure level (Table 9) and higher RWUE (3.573 kg ha^−1^mm^−1^) was observed from 15 t ha^−1^ cattle manure and Macia.

**Table 9:** Effects of cattle manure on RWUE of sorghum during 2017–2020.

Treatments	Mean Macia RWUE (kg ha^−1^mm^−1^)				Mean SV1 RWUE (kg ha^−1^mm^−1^)			
Cattle manure (t ha^−1^)	2017/18	2018/19	2019/20	Mean	2017/18	2018/19	2019/20	Mean
0	2.489^e^	2.628^e^	2.529^e^	2.55	2.208^e^	2.633^e^	2.455^e^	2.43
2.5	2.651^d^	2.872^d^	2.717^d^	2.75	2.415^d^	2.868^d^	2.638^d^	2.64
5	2.872^c^	3.046^c^	2.859^c^	2.93	2.82^c^	3.116^c^	2.894^c^	2.94
10	3.145^b^	3.261^b^	3.034^b^	3.15	3.073^b^	3.318^b^	3.16^b^	3.18
15	3.379^a^	3.573^a^	3.244^a^	3.4	3.438^a^	3.516^a^	3.313^a^	3.42
*P-value*	<0.001	*<0.001*	*<0.001*		*<0.001*	*<0.001*	*<0.001*	
LSD (0.05)	0.1225	0.0776	0.0196		0.1225	0.0776	0.0196	

Means in the same column followed by the same superscript (a-e) are not significantly different at p ≤ 0.05 using Fisher's Least significant difference.

### 4.4 Combined effects of RWH techniques, variety and cattle manure on RWUE

Interaction of RWH, season, cattle manure and sorghum variety show significant (p < 0.05) influence on RWUE (Table 10). Rainwater use efficiency show significant (p < 0.05) increase as application rates of cattle manure were increased (Table 10). Highest RWUE (3.885 kg ha^−1^ mm^−1^) was observed from TC + 15 t ha^−1^ cattle manure + Macia during 2018/19 cropping season. Sorghum variety, Macia had significant effects on RWUE at all rates of cattle manure across all cropping seasons. Lowest RWUE was observed from all SC treatments irrespective of season, variety and application rates of cattle manure (Table 10). Application of cattle manure integrated with RWH techniques show positive improvements on RWUE, with tied contour showing higher increments as application rates increases. Effects of combining RWH techniques with cattle manure significantly increased RWUE by 11.4–27.6 % (infiltration pits), 8.6–29.5 % (tied contours), 5.2–28.5 % (standard contours) over treatments without cattle manure.

**Table 10:** Combined effects of RWH, variety and cattle manure on RWUE of sorghum from 2017 to 2020.

Treatments	Cattle manure (t ha^−1^)	Mean Macia RWUE (kg ha^−1^mm^−1^)			Mean SV1 RWUE (kg ha^−1^mm^−1^)		
RWH techniques		2017/18	2018/19	2019/20	2017/18	2018/19	2019/20
Infiltration Pit	0	2.564^b^	2.658^b^	2.472^a^	2.179^a^	2.664^a^	2.436^b^
	2.5	2.623^bc^	2.997^de^	2.643^b^	2.341^ab^	2.915^b^	2.544^b^
	5	2.977^d^	3.105^de^	2.873^d^	2.8^d^	3.18^c^	2.852^cd^
	10	3.161^de^	3.349^f^	3.036^de^	3.108^de^	3.349^cd^	3.190^d^
	15	3.272^e^	3.539^g^	3.259^ef^	3.521^f^	3.519^d^	3.361^de^
Tied contour	0	2.612^b^	2.739^c^	2.761^c^	2.223^a^	2.726^ab^	2.649^bc^
	2.5	2.951^f^	2.997^de^	2.898^d^	2.590^c^	2.963^bc^	2.911^cd^
	5	2.977^d^	3.193^de^	2.99^de^	3.121^de^	3.268^cd^	3.082^cd^
	10	3.364^e^	3.403^f^	3.174^e^	3.161^e^	3.41^d^	3.311^de^
	15	3.738^f^	3.885^h^	3.357^f^	3.659^f^	3.661^d^	3.37^e^
Standard contour	0	2.289^a^	2.488^a^	2.354^a^	2.203^a^	2.508^a^	2.279^a^
	2.5	2.38^a^	2.624^b^	2.610^b^	2.315^ab^	2.725^ab^	2.459^b^
	5	2.662^bc^	2.841^cd^	2.715^c^	2.538^c^	2.902^b^	2.748^c^
	10	2.912^c^	3.031^de^	2.892^d^	2.951^de^	3.193^c^	2.977^cd^
	15	3.128^d^	3.295^e^	3.115^de^	3.134^e^	3.37^cd^	3.207^d^
LSD (5 %)		0.074	0.047	0.012	0.074	0.047	0.012
*P-value*		*<0.001*	*0.012*	*<0.001*	*0.001*	*<0.001*	*<0.001*
CV (%)		3.7	2.3	3.1	3.7	2.3	3.1

Means in the same column followed by the same superscript (a-h) are not significantly different at p ≤ 0.05 using Fisher's Least significant difference.

## 5 Discussion

### 5.1 Soil characterisation

Physiochemical properties of soil in the experimental plots were improved with the application of cattle manure which increased soil organic carbon, improve soil aggregation, water retention and reduce leaching of nutrients [7,41]. Application of organic nutrient source has residual effects in the soil which improve microbial population and soil physiochemical properties [35].

### 5.2 Grain yields

Rainfall received during the experimental period (2017/18 to 2019/20) was below the long-term average of 335 mm per cropping season in Chivi district [26]. Total amount of rainfall received per season qualifies the experimental site to be in agroecological region IV [13,45,46]. Rainfall received was associated with frequent droughts and this makes the area to be a semi-arid region. Climate change totally shifted onset of rainfall in Chivi compared to two decades ago where rainfall was received in mid-October, allowing early planting. This causes smallholder farmers in semi-arid areas of Zimbabwe to be vulnerable and face high risk of declining sorghum grain yield especially those who depend on rainfed agriculture. These challenges are associated with climate change and there is need for modifying farming systems to improve crop yields in smallholder farming environments. The use of improved sorghum varieties such as Macia, and adoption of TC and IP represents innovative and sustainable climate change adaptation strategy which improve food security in semi-arid areas [25]. Most rainwater is lost as runoff and little is harvested but the use of TC retains water in-field which can sustain crop growth and development during dry spells [11,14].

Sorghum variety Macia produced better grain yield under all RWH techniques compared with SV variety. Macia variety has been demonstrated to adapt well in semi-arid areas and produce better yield in Zimbabwe (0.4-0.8 t ha^−1^) and Tanzania (0.4–0.969 t ha^−1^) under water and nutrient management compared with other sorghum varieties [13,21,23,24]. This concurs with findings by Ref. [10] who reported higher sorghum grain yield from Macia variety than SC Sila under different RWH techniques. Tied contour and infiltration pits capture runoff water, making it available to crops during dry spell, and this improve crop growth and development. Availability of soil water reduce drought and nutrient stress, and improve crop yields [7,25]. The use of local varieties can be a better option to improve sorghum grain yield especially in smallholder farming environments due their better adaptation to local environmental conditions. This was in agreement with [47] who reported higher sorghum yield (2.411 t ha^−1^) in Nigeria than other varieties. This was the same scenario in semi-arid areas of Zimbabwe where sorghum grain yield is limited by low rainfall and soil infertility.

Application of cattle manure increase sorghum grain yield allowing farmers to meet their food demand of 800 kg per annum for a family of six. This concurs with [26] who reported that the use of organic nutrient sources improves food security and reduce poverty in smallholder farming environments. Animal manure improves soil health, reduce leaching of nutrients and can be used to achieve smart climate agriculture [10,41]. Application of cattle manure promotes plant physiological processes such as photosynthesis and protein synthesis which are crucial in achieving heavier grains [26]. Adoption of organic nutrient amendments has a potential of improving soil cation exchange capacity [48], decrease bulk density, improve plant nutrient availability and, boost plant growth and development [4,26,49,50]. Sorghum grain yield obtained from this study with application of cattle manure ranges from 0.822 to 1.09 t ha^−1^ showing 0.422–0.69 t ha^−1^ yield increment above 0.4 t ha^−1^ reported by Ref. [23] with the use of cattle manure.

Sorghum grain yield was improved with the use of RWH techniques, tied contours had higher yield compared with IP and SC. Tied contours used had higher water holding capacity compared with IP and SC. Tied contour recharge soil moisture, increasing water availability to crops. Results from this study were supported with findings by Ref. [2] who recorded higher crop yields from TC compared with IP and SC. This was also in agreement with results by Refs. [11,19] who obtained higher grain yield from TC in comparison with IP and SC. Sorghum grain yield was better during 2018/19 cropping season because of better rainfall distribution and residual effects of nutrient sources from previous season. These results were corroborating with findings by Ref. [21] who observed high sorghum grain yield from Macia variety during the same cropping season (2018/19) in Tanzania under in-situ RWH. Early planting in 2018/19 cropping season facilitates better utilisation of available soil moisture especially in January where sorghum was at flowering stage. Grain filling was done in February and facilitated by good rains received during the first two weeks of the month. Results from this experiment were related to findings by Refs. [7,26] who reported higher yields during same experimental seasons.

Combining RWH techniques and cattle manure improved sorghum grain yield across all treatments irrespective of season and variety. Integration of cattle manure with RWH techniques improves soil water retention capacity, availability of nutrients and increase sorghum grain yield [21,29]. Significant results reported from this study were supporting findings by Ref. [16] who reported that interactive effects of farm yard manure and in-situ RWH increased sorghum grain yield. Higher sorghum grain yield was observed from all treatments with TC. This was because tied contour retains a lot of water which can be used by plants [14,25]. The use of improved RWH techniques such as TC and IP can increase sorghum grain yield when integrated with organic nutrient sources such as cattle manure, Leucaena biomass and farm yard manure.

The results of this study show that the application of cattle manure significantly improved sorghum grain yield in the semi-arid area of Zimbabwe, with yields ranging from 0.67 to 1.146 t ha^−1^. The highest yield was obtained at the rate of 15 t ha^−1^ + TC + Macia, which is consistent with the findings of our previous study [13] that showed same sorghum grain yield at a combined rate of 15 t ha^−1^ of Leucaena/cattle manure combination under TC + Macia. Interestingly, the yields in this study are comparable to those obtained in the previous study ranging from 0.58 to 1.2 t ha^−1^ despite the different approaches used. This suggests that the combination of rainwater harvesting and cattle manure is as effective as the integration of *Leucaena leucocephala* and cattle manure (15 t ha^−1^) under rainwater harvesting techniques in improving sorghum productivity in semi-arid areas of Zimbabwe [13]. The comparison of the two studies also highlights the importance of optimal manure application rates. In this study, yield increment decreased at higher manure rates (15 t ha^−1^), suggesting that excessive manure application can be detrimental to sorghum productivity. Similarly, the previous study showed that yield increment decreased at combined rates of 20 t ha^−1^ and above of Leucaena leucocephala and cattle manure [13]. Conclusion from published study [13] showed that application of 10 t ha^−1^ Leucaena/cattle manure combination + TC + Macia variety gave sustainable sorghum grain yield and return on investment. This study also proved to provide sustainable sorghum grain yield at 15 t ha^−1^ cattle manure + TC + Macia. The differences in application rate were due to extra nitrogen (24 g kg^−1^ biomass above cattle manure) with the use of Leucaena biomass. However, it is better for farmers to choose the best nutrient source to use according to availability in their farming areas. These studies give an insight to framers on which application rate is sustainable when applying cattle manure or Leucaena/cattle manure combination. Farmers who are able to get Leucaena can combine it with cattle manure to produce 10 t ha^−1^ and those who get cattle manure only can use 15 t ha^−1^. Overall, the findings of this study and the previous study [13] demonstrate the potential of water conservation, soil fertility management, and crop management practices to improve sorghum productivity in semi-arid areas of Zimbabwe. However, optimal manure application rates and integration with other approaches like rainwater harvesting and legume intercropping are critical for achieving maximum yields.

### 5.3 Stover yield

Stover yield have been improved with the use of RWH techniques. Sorghum growth and development was improved with TC compared with IP and SC, this contributed to better photosynthetic rate which increase carbohydrate accumulation in stover. High capacity of water captured by TC also contributed to reduction in moistures stress, increased nutrient availability and higher leaf area index which facilitated photosynthesis and increase in stover yields. These results were in agreement to observations by Ref. [2] who reported higher growth rates and development using TC, causing improvements in stover yields. Application of cattle manure at different rates increased stover yields. This may be attributed to improved nutrient availability, reduced soil moisture stress and increased microbial population which facilitates decomposition making more nutrients available. These results were corroborating to findings by Ref. [13] who reported that application of Leucaena/cattle manure combinations increase stover yield. Stover yield from this study had same trend with increment in sorghum grain yield. This concur with results by Refs. [14,25,51] who reported that increase in sorghum grain yield can be transformed to higher stover yield. Combining RWH and cattle manure increased stover yield during the experimental period. The increments can be attributed to increased soil water content and availability of nutrients in plant rooting zone which promote plant growth and increase photosynthetic area [52,53]. This can be transformed into higher crop biomass leading to increments in stover yields. Findings from this study was related to research by Ref. [7] who reported increments in stover yield with the use of RWH and Leucaena biomass.

Observations from this experiment concur with results by Ref. [16] who indicated that use of RWH techniques and organic nutrient amendments increase water retention capacity, delay water depletion and increase sorghum yields. This also corroborates to research findings by Ref. [52] who observe increase in stover yield of pearl millet after applying 900–2700 kg ha^−1^ of cattle manure. These findings were concurring to related research by Refs. [54,55] who observed improvements in stover yield with increase in application rates of organic manure integrated with RWH techniques. Integrated nutrient management show a good potential in increasing sorghum stover yield. This was similar to related research by Ref. [56] who reported that combining mineral and organic fertiliser increased pearl millet stover yield by 121.12 % over control treatments.

### 5.4 Rainwater use efficiency (RWUE)

Macia had better RWUE compared with SV1 during the experimental period. This could be attributed to better adaptation of Macia variety to harsh conditions experienced semi-arid areas. These results were similar to findings by Ref. [21] who reported better RWUE from Macia than other local varieties, although the values were lower (1.06–2.13 kg ha^−1^ mm^−1^) than from this study in 2018/19 season. Tied contour had higher RWUE compared with IP and SC. These results were caused by increment in water capacity of runoff captured by TC which increased crop water available during dry period and improve crop yields. These values of RWUE were in agreement with [32] who observed RWUE of 2.1–3.2 kg ha^−1^ mm^−1^ with the use of open plough furrow in 1995/96 cropping season. The results from this study were related to findings by Ref. [7] who both observed RWUE of 2.2–3.49 kg ha^−1^ mm^−1^ with the use of TC and IP in sorghum during 2017/18 to 2019/20 cropping season. RWUE was lowest from SC because it dispose-off runoff water making it unavailable to crops during dry spell causing wilting and reduction in grain yield. Rainwater use efficiency was improved with increase in application rates of nutrient source. This concurs with report by Ref. [47] who concluded that increase in nutrient application rates increases crop water use efficiency. This was similar with reports by Refs. [7,54,55,57] who reported that increments in RWUE was caused by additional application of organic manure. This was agreed to be a result of improved soil structure, availability of micropores and reduced percolation of water by application of organic nutrient sources leading to increased crop yields. Significantly higher RWUE from tied contours combined with cattle manure show a balanced performance in soil moisture and nutrients availability to crops. This show that tied contours have the capacity to capture rainwater and cattle manure improves water retention capacity, hydraulic conductivity [48] which improves RWUE of sorghum. Results from this study corroborates with results by Refs. [55,58] who reported increments in RWUE of pearl millet from Zai pits + organic manure. Findings from this experiment concur with results by Ref. [52] who reported that modifying nutrient management and tillage practices improves nutrient availability and can increase RWUE by 25–40 %. These results were supported with findings by Refs. [57,59] who reported that the use of organic manure may improve RWUE in pearl millet although the values were lower compared to those from this experiment. RWUE from this experiment was in similar range with findings by Refs. [57,60] in Texas who observed a range of 1.5–5.5 kg ha^−1^mm^−1^ on sorghum. Observations from this study supported results by Ref. [21] whose RWUE values were ranging from 1.06 to 4.41 kg ha^−1^mm^−1^ for Macia variety with the use of in-situ RWH. The study managed to give better RWUE compared with findings from other semi-arid areas in Africa. This study was limited with long mid-season dry spells and continuous outbreak of fall armyworm which reduced crop performance. Lack of funding to cater for multiple research sites also limited the study to come up with better results which are more comparable across the country.

## 6 Conclusion

Results from this experiment demonstrate that TC can be an innovative and sustainable way of harvesting rainwater for future use in marginalised areas. Tied contour show yield benefits compared with other RWH techniques. However, results from this study show that TC can be best combined with cattle manure at 15 t ha^−1^ to improve sorghum yields and meet food demand in semi-arid areas. Increasing application rates of cattle manure show increments in grain and stover yields together with RWUE. Combining TC and cattle manure can be a smart climate agriculture option which is sustainable for smallholder farmers in semi-arid areas who are unable to buy irrigation equipment and adequate quantities of mineral fertiliser. Macia variety performed better than SV1 across all parameters over three cropping seasons. We can conclude that smallholder farmers can adopt the use of TC + 15 t ha^−1^ cattle manure + Macia variety to improve food availability, reduce food insecurity even during seasons when low rainfall (<335 mm) is received.

## Financial statement

No funding was available for this experiment.

## Data availability

Data included in article/referenced in the article.

## CRediT authorship contribution statement

**Andrew Tapiwa Kugedera:** Writing – review & editing, Writing – original draft, Visualization, Validation, Supervision, Resources, Project administration, Methodology, Investigation, Formal analysis, Data curation, Conceptualization. **Letticia Kudzai Kokerai:** Writing – review & editing, Writing – original draft, Investigation, Data curation, Conceptualization. **George Nyamadzawo:** Writing – review & editing, Writing – original draft, Supervision, Software, Resources, Formal analysis, Data curation, Conceptualization. **Ronald Mandumbu:** Writing – review & editing, Writing – original draft, Validation, Software, Methodology, Investigation, Formal analysis, Data curation, Conceptualization.

## Declaration of competing interest

The authors declare that they have no known competing financial interests or personal relationships that could have appeared to influence the work reported in this paper.
